# Maintaining Adherence to COVID-19 Preventive Practices and Policies Pertaining to Masking and Distancing in the District of Columbia and Other US States: Systematic Observational Study

**DOI:** 10.2196/40138

**Published:** 2023-04-25

**Authors:** Monica S Ruiz, Mercedes V McMahon, Hannah Latif, Amita Vyas

**Affiliations:** 1 Department of Prevention and Community Health Milken Institute School of Public Health George Washington University Washington, DC United States

**Keywords:** COVID-19, mask adherence, social distancing, public health, health policy, public health mandates

## Abstract

**Background:**

Prior to the development of effective vaccines against SARS-CoV-2, masking and social distancing emerged as important strategies for infection control. Locations across the United States required or recommended face coverings where distancing was not possible, but it is unclear to what extent people complied with these policies.

**Objective:**

This study provides descriptive information about adherence to public health policies pertaining to mask wearing and social distancing and examines differences in adherence to these policies among different population groups in the District of Columbia and 8 US states.

**Methods:**

This study was part of a national systematic observational study using a validated research protocol for recording adherence to correct mask wearing and maintaining social distance (6 feet/1.83 meters) from other individuals. Data were collected from December 2020 to August 2021 by research team members who stationed themselves in outdoor areas with high pedestrian traffic, observed individuals crossing their paths, and collected data on whether individuals’ masks were present (visible or not visible) or worn (correctly, incorrectly, not at all) and whether social distance was maintained if other individuals were present. Observational data were entered electronically into Google Forms and were exported in Excel format for analysis. All data analyses were conducted using SPSS. Information on local COVID-19 protection policies (eg, mask wearing requirements) was obtained by examining city and state health department websites for the locations where data were being collected.

**Results:**

At the time these data were collected, most locations in our study required (5937/10,308, 57.6%) or recommended (4207/10,308, 40.8%) masking. Despite this, more than 30% of our sample were unmasked (2889/10136, 28.5%) or masked incorrectly (636/10136, 6.3%). Masking policy was significantly related to correct masking with locations that required or recommended masking (66% correct masking vs 28/164, 17.1% in locations that did not require masking, *P*<.001). Participants who maintained social distance from others were more likely to be correctly masked than those who were not (*P*<.001). Adherence to masking policy by location was significant (*P*<.001); however, this was driven by 100% compliance in Georgia, which did not require masks at any point during the data collection period. When the same analysis was conducted for compliance with mask requirements and recommendations, there was no significant difference by location. Overall adherence to masking policies was 66.9%

**Conclusions:**

Despite a clear relationship between mask policies and masking behavior, one-third of our sample was nonadherent to those policies, and approximately 23% of our sample did not have any mask, either on or visible. This may speak to the confusion surrounding “risk” and protective behaviors, as well as pandemic fatigue. These results underscore the importance of clear public health communication, particularly given variations in public health policies across states and localities.

## Introduction

Since the novel COVID-19 pandemic became known in January 2020, the United States has experienced over 103 million cases and over 1.1 million deaths (cumulative as of February 25, 2023) [1]. From the beginning of the pandemic, certain communities have been shown to be disproportionately affected by COVID-19; a recent systematic review of disparities in COVID-19 infections, hospitalizations, and deaths showed that African Americans/Blacks and Hispanic/Latinx populations had disproportionately higher rates of SARS-CoV-2 infection, hospitalization, and COVID-19–related mortality compared with non-Hispanic/Latinx White populations [2]. Other data show that older age and presence of health comorbidities—including presence of obesity and diabetes, cardiovascular disease, and chronic kidney disease—are associated with higher risk of COVID-19 morbidity and mortality [3].

Although the development and widespread dissemination of effective vaccines to prevent COVID-19 infection have helped to reduce infection rates, voluntary uptake of the vaccine has been slower than expected. National surveillance data show that, although 81.1% of the total US population—including 92% of adults over the age of 18 years—have had at least 1 dose of the SARS-CoV-2 vaccine, only 69.2% completed the primary series and only 16% have received an updated bivalent booster dose [4]. Nonetheless, vaccine uptake, combined with continued preventive behaviors (ie, mask wearing and social distancing), remain the most effective ways to minimize infection and transmission risk [5-9].

The importance of mask wearing continues to be an effective way to protect against infection against COVID-19 and other respiratory infections. There have been numerous studies of the effectiveness of mask wearing against SARS-CoV-2 infection. For example, in a cross-sectional, web-based study of US-based individuals, Rader et al [10] found that a reported 10% increase in mask wearing was associated with more than a 3-fold increase in infection control and that communities with high reported mask wearing and social distancing had the highest predicted probability of transmission control. In another study from Japan that surveyed close contacts of SARS-CoV-2–infected patients, Sugimura et al [11] found that the individuals who indicated that they did not wear face masks were infected at a higher rate than mask wearers (16.4% compared with 7.1%). A comprehensive narrative review published in 2021 by Howard et al [12] found support for the effectiveness of public mask wearing, stating that “...public mask wearing is most effective at reducing spread of the virus when compliance is high...in conjunction with existing hygiene, distancing, and contact tracing strategies.” They further recommended that “public officials and governments strongly encourage the use of widespread face masks in public, including the use of appropriate regulation” [12]. The importance of continued masking is underscored by the presence of highly transmissible variants such as the Omicron variants, which, combined, accounted for 93.8% of infections in the United States (as of February 25, 2023) [13] and are contagious even among those who are fully vaccinated and boosted.

The issue of regulation—such as the enforcement of vaccine and mask wearing mandates—for the protection of public health has historically been and continues to be contentious [14]. In the way the COVID-19 pandemic unfolded in the United States, general vaccine hesitancy, politicization of governmental mandates, and the politicization of the pandemic itself served to hamper timely uptake and adherence to public health recommendations for mask wearing at a time when infections were rapidly spreading and vaccines had not yet become available. The politicization of the pandemic and mask wearing by conservative governmental leaders was particularly noteworthy; in the absence of a national mask wearing mandate, individual states implemented legislation requiring the wearing of face masks in public. In August 2020, while the epidemic was sweeping through the United States, 34 states and the District of Columbia had mask mandates, while 16 states—each of which had a Republican governor—did not [14]. A study that examined adherence to state-level masking policies and SARS-CoV-2 infection rates in those states at around this same time period in 2020 found that 93% of the states that had no mask wearing policies for the general public reported high COVID-19 rates, whereas none of the 8 states that had at least 75% adherence to masking policies reported high infection rates [15].

The effectiveness of a public health mandate to protect against disease spread may also be dependent on the level of mobilization of people in and and out of the community. For example, efforts to prevent SARS-CoV-2 infection and transmission in metropolitan regions may be especially tricky given the movement of people into and out of cities and their surrounding suburban areas, and Black and Indigenous People of Color (BIPOC) communities may have elevated levels of vulnerability to COVID-19 because of their participation in sectors of the workforce that are deemed essential. For example, the pandemic picture in the Washington, DC, region—which includes the District of Columbia and immediate surrounding jurisdictions in Maryland and Virginia—mirrors that of the rest of the nation in terms of its impact on disproportionately affected populations [16-18]. Because of its location between Maryland and Virginia, the District has very fluid boundaries and experiences a significant amount of population migration to and from these states as people enter and exit the District for employment, commerce, and tourism. As a result, the level of infection risk for SARS-CoV-2 in the District may be affected by factors in its neighboring jurisdictions, including the R0 (basic reproduction number), rate of vaccination uptake, and levels of social and community vulnerability that might increase SARS-CoV-2 infection risk. For these reasons, adherence to preventive behaviors in urban centers is of paramount importance.

Although there are data about the adherence to these preventive behaviors for other locations in the country, little is known about the levels of adherence to these behaviors in the metropolitan Washington, DC area and how this region compares with other metropolitan centers. The purpose of this paper was to provide descriptive information about adherence to mask wearing and social distancing in the District of Columbia and 8 other US states and to examine differences in adherence among different population groups within these locations.

## Methods

This study was conducted by the George Washington University team that was part of the larger, national-level Systematic Observation of Mask Adherence and Distancing (SOMAD) study [19]. Although the methodological details pertaining to this study are provided elsewhere [20], we will also explain them in the following sections to provide clarity about the study procedures.

### Student Researcher Training

The research team included 13 student researchers who were trained by the SOMAD principal investigator (via Zoom meeting) on the study protocol [21] and data collection procedures. The student researchers spent approximately 1 week practicing the data collection procedures to ensure comfort with and ability to adhere to the study protocol. Issues that arose during the “practice data collection” sessions were discussed with our university’s faculty team leaders to ensure that study procedures were clear and that data collection practices were adherent with the master SOMAD protocol. Student researchers were advised that, although it may be difficult to ascertain exact ages, genders, and race/ethnicity of persons observed, they should use their best judgment based on the way the person is presenting.

### Data Collection Methods

Our study was conducted from December 1, 2020, to August 1, 2021. During this time, the university was operating virtually, so student researchers were attending classes and research team meetings from their home locations. Student researchers chose places close to home as their observation sites. Although most data were collected in the metropolitan Washington, DC area (which includes parts of Maryland and Northern Virginia), we did have team members who were collecting data in other states. As such, in addition to data from the metropolitan Washington, DC area, data were collected from Georgia, Missouri, Arizona, California, Nevada, and Illinois.

As these data were collected prior to the wider availability of COVID-19 vaccines, all observation locations were outdoors and in high pedestrian traffic areas, such as parks, multi-use paths and trails (eg, walking, running, and biking trails), city intersections, and shopping areas that had outdoor pedestrian walkways and spaces. Student researchers wore masks and maintained social distance from others while collecting data to protect themselves from infection risk.

Per the SOMAD protocol [21], the student researcher (acting as the observer) selected a specific spot for data collection. As individuals passed by that spot, the student researcher recorded the characteristics of that person, entered those data into the online data collection form, and then continued to observe and enter data for the next person who passed by. If persons were walking in groups, student researchers would assess the general characteristics of 4 persons within each group (in order to retain as much detailed information as possible) and enter those data into the online form for each person.

Data were collected for a minimum of 2 hours per week, with 1 observation session occurring on a weekday and 1 session occurring on a weekend. Both observation sessions occurred at the same time of day to ensure comparability between weekdays and weekends. Data were collected using the national SOMAD team’s online data collection form. Data were then cleaned by the national team’s statistician and returned to our university team for analyses.

### Measures

Data were collected pertaining to a variety of characteristics related to the outdoor space where data collection occurred and the persons being observed. These variables are described in the following sections.

#### Location and Setting Variables

At the start of each observation session, the student researcher recorded the date, time, city, address, and zip code in which the observation was occurring. Additionally, student researchers recorded the type of location where the observation was occurring (eg, commercial street, neighborhood park, trail) and, if applicable, the official name of the location (eg, the specific name of the park or trail). Student researchers also noted if the location had a masking policy and whether there was sufficient space in the location for social distancing, which is defined as being able to maintain 6 feet or 1.83 meters from another person.

#### Demographic Variables

With regard to the persons being observed, it was understood that it is often difficult to ascertain with certainty the demographic characteristics of individuals simply by looking at physical characteristics. Such judgments may be made even more difficult if individuals are wearing face coverings. Nonetheless, student researchers were asked to do the best they could to ascertain the approximate age, gender, and race/ethnicity of the persons being observed. Options for age group included infant or toddler (0-2 years of age), child (3-12 years of age), adolescent or teen (13-19 years of age), adult (20-59 years of age), and older adult (60 years of age and older). With regard to gender and race/ethnicity, student researchers were asked to note the gender (male, female, unable to judge) and apparent race/ethnicity (White, Black/African American, Asian, Latinx, unable to determine) of each observed individual.

Additionally, as these observations were occurring in outdoor spaces, student researchers were asked to record the physical activity levels of the individuals. Individuals could be categorized as being sedentary (eg, sitting, laying down, or being carried in the case of a child), moderately active (eg, walking or biking slowly), or vigorously active (eg, running, biking, climbing). Similarly, individuals using modes of transportation were recorded as being either “on wheels” (ie, being on a bicycle or skateboard or in a wheelchair or stroller) or “not on wheels” (ie, being on foot).

#### COVID-19 Protective Practices

Student researchers collected data on the adherence to COVID-19 protective practices of mask wearing and social distancing. For mask wearing, individuals were noted as having their masks on correctly (ie, covering the nose and mouth), wearing their masks incorrectly (eg, mask covering mouth but not nose or vice versa), having visible masks present but not on their face (eg, masks in hand, dangling from an ear, or being worn under the chin), or having no face covering at all. With regard to social distancing, student researchers recorded if observed individuals were traveling alone or in a group (defined as 2 or more people together) and whether individuals (including those traveling in groups) were maintaining social distance from others.

#### Policy Variables

In addition to the notation about masking policies of specific locations where observations occurred, we collected data pertaining to state and local masking and social distancing policies through examination of city and state health department websites for the locations where data were being collected.

### Analyses

Observational data were entered electronically into Google Forms and were then exported in Excel format for analysis. Univariate and bivariate analyses were conducted to obtain descriptive information about the observational sample. Pearson’s chi-square analyses were used to test for significant associations between study variables. All data analyses were conducted using SPSS version 24.

### Ethical Considerations

Prior to engaging in data collection, the study protocol was submitted to the George Washington University’s Institutional Review Board for review. A waiver of informed consent was requested due to (1) the nature of the study being purely observational and not requiring personal interaction with prospective participants (ie, those persons being observed) and (2) the fact that no protected personally identifying information or protected health data were collected from individuals who were observed as part of the study. Because no personally identifying information were collected, all data from persons observed were anonymous and analyzed in aggregate. Finally, as these data were collected without involving interpersonal interaction, individuals whose behaviors were observed as part of this study were not offered compensation. Based on these aspects of the study, the George Washington University Institutional Review Board determined that this study constituted minimal risk to research participants and therefore was exempt from review (IRB# NCR213396).

## Results

### Sample Demographics

**Figure 1:**
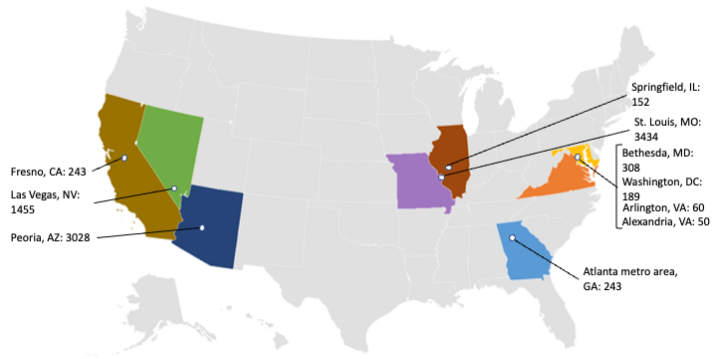
Study locations and daily average new COVID-19 cases (as of December 2020).

The findings presented here represent data collected between November 29, 2020, and August 1, 2021, in multiple locations across 8 states and the District of Columbia. The geographic locations of our study sites in the United States are shown in Figure 1. In addition to geographic location, we have provided the daily average of new COVID-19 cases in each location (using data from the New York Times COVID-19 Tracker) [22] in order to demonstrate how the pandemic was affecting each state or, where more specific data were available, each city at the approximate date when data collection started.

Demographic data about the individuals observed are presented in Table 1. A total of 10,308 observations were collected. The sample was relatively evenly split between persons observed to be male and persons observed to be female (4539/10,081, 45% vs 5542/10,081, 55%), with most persons observed being adults between the ages of 20 years and 59 years (7490/10,118, 74%), of White or Black race (5271/10,308, 51.1% and 2623/10,308, 25.4% respectively), and of non-Latinx ethnicity (8928/9822, 90.9%). Of the observations, 47% (4836/10,291) were collected in the metropolitan Washington, DC area, and 38% (3942/10,291) came from 3 sites in the metropolitan Atlanta area in Georgia. Most data collection locations had policies requiring (5937/10,308, 57.6%) or recommending (4207/10,308, 40.8%) masking in outdoor locations.

**Table 1:** Sample demographics (n=10,308).

Variable			Results^a^, n (%)
**Sex^b,c^**			
	Male	4539 (45)	
	Female	5542 (55)	
**Age^b,d^ (years)**			
	0-2	110 (1.1)	
	3-12	542 (5.4)	
	13-19	802 (7.9)	
	20-59	7490 (74)	
	≥60	1174 (11.6)	
**Race^b^**			
	White	5271 (51.1)	
	Black	2623 (25.4)	
	Asian	1034 (10)	
	Other/unknown^e^	1380 (13.4)	
**Ethnicity^b,f^**			
	Latinx	894 (9.1)	
	Non-Latinx	8928 (90.9)	
**Data collection location^g^**			
	Washington, DC	858 (8.3)	
	Alexandria, VA	1578 (15.3)	
	Arlington, VA	660 (6.4)	
	Bethesda, MD	1740 (16.9)	
	Atlanta, GA	167 (1.6)	
	Lawrenceville, GA	3628 (35.3)	
	Dunwoody, GA	147 (1.4)	
	Las Vegas, NV	1201 (11.7)	
	Peoria, AZ	12 (0.1)	
	Springfield, IL	180 (1.7)	
	St Louis, MO	90 (0.9)	
	Fresno, CA	30 (0.3)	
**Outdoor masking policy**			
	Mask required	5937 (57.6)	
	Mask recommended	4207 (40.8)	
	Mask not required	164 (1.6)	

^a^The total sample sizes for the variables may not match due to missing data.

^b^Indicated by observer report based on appearance.

^c^n=10,081.

^d^n=10,118.

^e^Observations marked as unknown have been combined with missing.

^f^n=9822.

^g^n=10,291.

### Adherence to COVID-19 Preventive Behaviors

Regarding adherence to social distancing, the sample was almost evenly split between those correctly social distancing (maintaining a distance between persons of >6 feet/1.83 meters; 5185/10,113, 51.3%) and those incorrectly social distancing (standing <6 feet//1.83 meters from others; 4928/10,113, 48.7%). Approximately 65% (6611/10,136, 65.2%) of participants observed were wearing masks correctly (ie, fully covering the nose and mouth), while another 23.2% (2353/10,136) were not wearing masks at all. These data are shown in Table 2.

When examining correlates of correct masking behavior, several interesting findings emerged. These data are presented in Table 3. Persons who were correctly masked were more likely to be those observed as female (3828/5538, 69.1%; *χ*^2^_1_=74.8, *P*<.001), of Asian race (793/1033, 76.8%; *χ*^2^_2_=129.6, *P*<.001), and aged 60 years or older (790/1172, 67.4%; *χ*^2^_4_=206.7, *P*<.001). Those correctly masked were also more likely to be adherent to social distancing guidelines (3556/5180, 68.6%; *χ*^2^_1_=55.7, *P*<.001).

Correct masking behavior was more likely to be observed outside of the metropolitan Washington, DC area compared with within the metropolitan Washington, DC area (3585/5401, 66.4% vs 3018/4718, 64%; *χ*^2^_1_=6.4, *P*=.01) and in locations with required or recommended outdoor masking policies (3796/5791, 65.5% and 2787/4181, 66.7%, respectively; *χ*^2^_2_=171.7, *P*<.001). We further examined potential differences in adherence to masking policy by state or region, focusing on the 4 states and regions for which we had the most data: the metropolitan Washington, DC area; Georgia; Nevada; and Illinois. These data are presented in Table 4. In the time during which these data were collected, the metropolitan Washington, DC area; Illinois; and Nevada had policies that mandated masking in both indoor and outdoor locations, while Georgia did not have a mask mandate in place. However, despite the absence of a state-level mask mandate, the businesses in Georgia where those data were collected did require or recommend that customers entering those indoor spaces wear masks. When we examined masking behavior and adherence to state policies where masking was mandated, we found that, overall, there was significant adherence to masking policies (*χ*^2^_3_=1584.1, *P*<.001), and the significance was largely driven by Georgia’s 100% adherence to a state policy that did not require masking. We then examined masking behavior and adherence to policies where masking was either mandated (eg, state policy) or required or recommended (eg, business policies in GA). When combining these 2 conditions, we found that, although overall levels of masking adherence were close to 66%, the comparison was no longer statistically significant (*χ*^2^_3_=3.2, *P*=.37).

**Table 2:** COVID-19 preventive behaviors among the study sample (n=10,308).

Variable			Results^a^, n (%)
**Social distancing behavior^b^**			
	Correctly social distancing	5185 (51.3)	
	Incorrectly social distancing	4928 (48.7)	
**Masking behavior^c,d^**			
	Mask on	6611 (65.2)	
	Mask partially on (eg, worn below nose)	636 (6.3)	
	Mask visible	536 (5.3)	
	No mask	2353 (23.2)	

^a^The total sample sizes for the variables may not match due to missing data.

^b^n=10,113.

^c^Categories are mutually exclusive.

^d^n=10,136.

**Table 3:** Correlates of correct masking behavior (n=10,136).

Variable			Correct masking, n (%)		Incorrect or no masking, n (%)		*χ*^2^ (*df*)		*P* value
**Location^a^**							6.4 (1)		.01
	Metropolitan Washington, DC area	3018 (64)		1700 (36)					
	Outside of Metropolitan Washington, DC area	3585 (66.4)		1816 (33.6)					
**Sex^b,c^**							74.8 (1)		<.001
	Male	2762 (60.9)		1774 (39.1)					
	Female	3828 (69.1)		1710 (30.9)					
**Race^c,d^**							129.6 (2)		<.001
	White	3194 (60.6)		2074 (39.4)					
	Black	1820 (69.4)		802 (30.6)					
	Asian	793 (76.8)		240 (23.2)					
**Ethnicity^c,e^**							1.7 (1)		.20
	Latinx	562 (62.9)		331 (37.1)					
	Non-Latinx	5807 (65.1)		3116 (34.9)					
**Age^f^ (years)**							206.7 (4)		<.001
	0-2	9 (8.2)		101 (91.8)					
	3-12	283 (52.8)		259 (47.8)					
	13-19	528 (65.8)		274 (34.2)					
	20-59	4983 (66.6)		2503 (33.4)					
	≥60	790 (67.4)		382 (32.6)					
**Distancing^g^**							55.7 (1)		<.001
	Correctly social distancing	3556 (68.6)		1624 (31.4)					
	Incorrectly social distancing	3033 (61.6)		1893 (38.4)					
**Mask policy**							171.7 (2)		<.001
	Required	3796 (65.5)		1995 (34.5)					
	Recommended	2787 (66.7)		1394 (33.3)					
	Not required	28 (17.1)		136 (82.9)					

^a^n=10,119.

^b^n=10,075.

^c^Unknown/other has been designated as missing.

^d^n=8923.

^e^n=9816.

^f^n=10,112.

^g^n=10,106.

**Table 4:** Adherence to masking policy by state or region.

State or region		Nonadherence with masking policy, n (%)	Adherence with masking policy, n (%)	*χ*^2^ (*df*)		*P* value	
**Policy: masking mandated**					1584.1 (3)		<.001
	Metropolitan Washington, DC area	1540 (32.6)	3178 (67.4)				
	Georgia	0 (0)	3942 (100)				
	Nevada	377 (31.4)	823 (68.6)				
	Illinois	34 (22.2)	119 (77.8)				
	Total	1951 (19.5)	8062 (80.5)				
**Policy: masking required or recommended**					3.2 (3)		.37
	Metropolitan Washington, DC area	1588 (33.7)	3130 (66.3)				
	Georgia	1300 (33.2)	2619 (66.8)				
	Nevada	377 (31.4)	823 (68.6)				
	Illinois	44 (33.1)	106 (66.9)				
	Total	3309 (33.1)	6678 (66.9)				

## Discussion

### Principal Findings

Our data show that, in a sample that included observations from 8 states and the District of Columbia, individuals who were observed to be female, older than 60 years, and of Asian race were more likely to be correctly adherent to public health guidelines in terms of the correct utilization of face masks when outdoors in public spaces. Moreover, we found that those who had engaged in correct masking behavior were also more likely to be adherent to social distancing guidelines. The findings from this study support the findings from the other SOMAD studies that also observed higher levels of adherence to correct mask usage among female individuals and persons of older age, as well as persons of Asian ancestry [20,23].

### Comparisons With Prior Work

It is of interest to note that, while most of the data collection sites had policies in place that either required or recommended masking outdoors, we found that approximately 23% of the sample were not wearing masks at the time they were observed. This may be due to the perception—which is supported by epidemiological data—that SARS-CoV-2 transmission risk is significantly lower in outdoor spaces [24]. To that point, we did find a 66% adherence to state or local masking policies. This finding supports the results of another national-level systematic observation study that found that mask mandates result in a 3-fold increase in adherence to mask wearing [25]. Additionally, our finding showed that local policies—such as those implemented by private businesses in Georgia requiring or recommending mask wearing by their customers even though there was no mask mandate in place at the state level—may be influential in prompting preventive behaviors even in broader environmental and legislative contexts where such behaviors are not deemed important. Together, these findings support the idea that policies promoting or mandating public health protective behaviors can be useful in helping states and localities mitigate the impact of disease spread. It is likely that future observational studies of indoor mask wearing may show different levels of adherence to ongoing recommendations pertaining to social distancing and mask wearing, particularly in light of rising rates of vaccination among the general population, changing state and local policies pertaining to indoor mask usage, and waning rates of infection in the general population.

### Limitations

There are several significant limitations of this research that should be noted. As mentioned previously, all data collected were observational. Because most participants were masked, it may have been more difficult than it would be under normal circumstances to ascertain persons’ race, ethnicity, sex, and age. Similarly, because of the diversity of people observed and general difficulties in guessing people’s ages, race/ethnicity, and gender, there is likely some amount of error in our data pertaining to our participants’ demographic characteristics. This lack of precision is a limitation of the data collection procedures. Also, because data collectors were frequently observing individuals as they moved through spaces (eg, crossing a street or walking on a sidewalk), it may be possible that observers were unable to clearly determine individuals’ demographic characteristics or mask usage. This may have contributed to misclassification of individuals into demographic and behavioral adherence categories.

### Conclusions

Despite these limitations, we were able to collect a substantial amount of data from a variety of locations across the country, thereby providing a representative picture of COVID-19 preventive behaviors in a diverse population. These data provide some significant takeaways that can inform public health practice as the world moves into the third year of addressing the COVID-19 pandemic. The first of these points is that, despite significant gains in vaccination uptake, there is still a need for adherence to COVID-19 preventive behaviors, particularly in places (eg, rural communities) and among populations (eg, communities of color) where disparities in vaccine access and uptake exist and where the presence of significant health comorbidities increase risk of severe disease if persons do become infected. Similarly, with the continued emergence of new SARS-CoV-2 variants, maintaining high levels of adherence to protective behaviors will be critical to preventing further surges in the numbers of COVID-19 cases, including both new and breakthrough infections. As the pandemic becomes endemic and more normalized, maintaining adherence to preventive behaviors is challenging, as the presence of effective vaccines may cause the public to believe that the pandemic is over or that preventive behaviors are no longer relevant. Future research should focus on developing more innovative ways to promote continued adherence to masking and social distancing guidance where necessary and appropriate, with particular attention to how to mitigate “pandemic fatigue” [26].

Our findings also highlight the importance of clear and effective public health communication regarding the need for adherence to preventive behaviors other than or in addition to vaccination. This is particularly necessary considering variations in state and local adherence with federal guidance, as well as the abundance of misinformation about COVID-19 that continues to circulate in the news media and on social media. A recent Kaiser Family Foundation report found that, in a sample of 1519 adults 18 years of age and older sampled through random digit dialing, approximately 78% indicated that they believed or were unsure about at least one false statement about the COVID-19 pandemic or about vaccination that they had seen in the news media [27]. Future research should focus not only on improving the health messages that are disseminated to the public about COVID-19 and disease prevention but also on improving health literacy among the general population so that individuals are better able to determine the veracity of information they hear through the news media so that they can more easily spot “fake news” about the pandemic. Moreover, given the continued evolution of highly transmissible and contagious SARS-CoV-2 variants, it is critical that public health campaigns effectively communicate the continued benefit of protective behaviors such as vaccination, masking, and distancing as strategies to reduce the morbidity and mortality associated with the COVID-19 pandemic.

## Abbreviations

BIPOC: Black and Indigenous People of Color
R0: basic reproduction number
SOMAD: Systematic Observation of Mask Adherence and Distancing

## Data Availability

The data sets generated or analyzed during the current study are available from the corresponding author (MSR) on reasonable request. Data requests for the national SOMAD study should be made to Dr. Deborah A Cohen (Deborah.A.Cohen@kp.org).
